# Discrepancies in Infectivity of Flavivirus and SARS-CoV-2 Clinical Samples: An Improved Assay for Infectious Virus Shedding and Viremia Assessment

**DOI:** 10.3390/ijerph18189845

**Published:** 2021-09-18

**Authors:** Mizuki Fukuta, Co Thach Nguyen, Thi Thu Thuy Nguyen, Thi Thanh Ngan Nguyen, Thi Bich Hau Vu, Taichiro Takemura, Le Khanh Hang Nguyen, Shingo Inoue, Kouichi Morita, Thi Quynh Mai Le, Futoshi Hasebe, Meng Ling Moi

**Affiliations:** 1 Graduate School of Biomedical Sciences, Nagasaki University, Nagasaki 852-8523, Japan; mixgoldenretriever@gmail.com (M.F.); thanhngan0605@gmail.com (T.T.N.N.); taichiro@nagasaki-u.ac.jp (T.T.); pampanga@nagasaki-u.ac.jp (S.I.); moritak@nagasaki-u.ac.jp (K.M.); rainbow@nagasaki-u.ac.jp (F.H.); 2 National Institute of Hygiene and Epidemiology, Hanoi 10000, Vietnam; thachnc2000@gmail.com (C.T.N.); ticun_2002@yahoo.com (T.T.T.N.); hauvtb@gmail.com (T.B.H.V.); nlkh@nihe.org.vn (L.K.H.N.); lom9@hotmail.com (T.Q.M.L.); 3 World Health Organization Collaborating Center for Reference and Research on Tropical and Emerging Virus Diseases, Institute of Tropical Medicine, Nagasaki University, Nagasaki 852-8523, Japan; 4 Graduate School of Medicine, The University of Tokyo, Tokyo 113-0033, Japan

**Keywords:** infectivity assessment, infectious particles, dengue, SARS-CoV-2, culture medium

## Abstract

Infectivity and neutralizing antibody titers of flavivirus and severe acute respiratory syndrome coronavirus 2 (SARS-CoV-2) are frequently measured using the conventional plaque assay. While the assay is useful in the determination of infectivity, conventional plaque assays generally possess lower sensitivity and are time-consuming compared to nucleic acid amplification tests. In this study, a microcrystalline cellulose (MCC), Avicel, was evaluated as an alternative to the conventional virus overlay medium, methylcellulose, for a plaque assay. The plaque assay was performed using dengue and COVID-19 clinical samples and laboratory-established flavivirus and SARS-CoV-2 strains. In virus titration of clinical samples, the plaques were significantly larger, and the virus titers were higher when Avicel MCC-containing overlay medium was used than with conventional methylcellulose overlay medium. In addition, for some clinical samples and laboratory virus strains, infectious particles were detected as plaques in the Avicel MCC-containing medium, but not in the conventional methylcellulose medium. The results suggest that the viremia titer determined using the new overlay medium containing Avicel MCC may better reflect the innate infectious and plaque-forming capabilities of clinical samples and better reflect virus infectivity.

## 1. Introduction

Virus shedding during acute viral infection has important implications for the transmission and control of infectious disease [1]. In addition to control measures, high blood viral load levels have been associated with greater disease severity, and an understanding of the detailed patterns of virus shedding and viremia is important for determining disease pathogenesis [2,3,4,5]. While quantitative real-time polymerase chain reaction (RT-PCR) is the gold standard for virus particle titration, the method detects viral nucleic acid but not the infectious capability of virus particles [6,7,8]. The infectivity of a virus is typically determined using permissive cells on which the virus has a cytopathic effect (CPE) or leads to plaque formation on virus infection [9,10]. Measuring the viral titer is further challenged if the clinical virus strain does not have a CPE or lead to plaque formation; hence, measurement of infectivity is limited to the detection of CPE forming viruses. As a result of the reliance on detection of a CPE, the infectivity may not be accurately reflected.

During the course of SARS-CoV-2 infection, individuals may remain PCR-positive for several weeks [11]. Early diagnosis is important for managing cases and controlling disease spread. While PCR positivity alone is not associated with infectiousness, accurate reflection of virus infectivity in association with virus shedding, as determined by RT-PCR, is an important consideration of test-based clearance and to accurately determine infectiousness [12]. To determine virus infectivity, conventional methods rely on CPE and plaque formation, and it is possible that these assessment methods may lead to discrepancies in measuring infectious virus titers because they have lower sensitivity than RT-PCR [13]. An ideal detection method should retain the simplicity and practical attributes of the standard virus detection method and demonstrate improved efficiency, reproducibility, shorter assay time, and adaptability to an array of viral infectious diseases. In the present study, we used flavivirus and SARS-CoV-2 clinical isolates and patient samples to determine the utility of an improved virus infectivity assessment method for virus titration using an improved overlay medium [14,15,16]. The novel method led to improved infectious virus detection and a higher virus titer compared to the conventional plaque assay. Infectious dengue virus (DENV) and SARS-CoV-2 were detected in some samples, despite negative results using conventional methods. Overall, the modified infectious virus particle detection system using Avicel microcrystalline cellulose [17] is simple and retains the practical features of conventional methods for the detection of infectious virus particles.

## 2. Materials and Methods

### 2.1. Cell Lines

Baby hamster kidney cells (BHK-21, Japan Health Science Research Resource Bank), African green monkey kidney Vero cells (Vero 9013, Japan Health Science Research Resource Bank), and FcγRIIA-expressing BHK-21 [18] were used in this study. BHK-21 and Vero cells were cultured in Eagle’s Minimum Essential Medium (E-MEM; Sigma, St. Louis, MO, USA) supplemented with heat-inactivated 10% fetal bovine serum (FBS, Sigma) without antibiotics at 37 °C in 5% CO_2_. FcγRIIA-expressing BHK-21 cell lines were cultured in E-MEM supplemented with heat-inactivated 10% FBS and 0.5 mg/mL neomycin (G418; PAA Laboratories GmbH, Pasching, Austria) at 37 °C in 5% CO_2_ [19].

### 2.2. Virus

The following viruses and strains were used in this study: DENV-1 (01-44-1 HuNIID and 99St12A strains); DENV-2 (DHF0663, TLC-30, 08-77, and 00St22A strains); DENV-3 (CH53469 strain); DENV4 (SLMC318 and TVP360 strains); Zika virus (ZIKV; MR766, PRVABC59, MRS_OPY_Martinique_PaRi_2015, and H/PF/2013 strains); Japanese encephalitis virus (JEV; OH0566 strain); yellow fever virus (YFV; 17D vaccine strain); SARS-CoV-2 (2019-nCoV/Japan/TY/WK-521/2020, NGS1B, hCoV-19/Japan/QK002/2020, hCoV-19/Japan/TY7-501/2021, and hCoV-19/Japan/TY8-612/2021 strains). Laboratory virus strains were propagated on Vero and BHK cells at 37 °C in 5% CO_2_ for 5–7 days or until CPE formation was observed. Cell culture supernatant was then collected, clarified by centrifugation, aliquoted and, stored at −80 °C until use.

### 2.3. Patient Samples

A total of 27 serum samples obtained from DENV-1 patients during a DENV outbreak in Northern Vietnam in 2017 were used in this study. All patients were confirmed to be infected with DENV-1 by RT-PCR [20] (Supplementary Information). DENV primary and secondary infections were distinguished by DENV immunoglobulin M (IgM) and immunoglobulin G (IgG) enzyme-linked immunosorbent assay (ELISA) (Vircell, Granada, Spain), and RT-PCR. A DENV E/M protein-specific IgM/IgG ratio was used to define primary and secondary infections. In primary infection, IgM levels increase the first day after virus infection, and IgG levels increase 4–5 days after infection. In the secondary infection, because of the presence of pre-existing immunity, IgG levels are higher during the early acute phase [21]. Primary infection was defined as samples with an IgM/IgG optical density (OD) ratio of ≥1.2, or negative for both DENV IgM and DENV IgG. Secondary infection was defined as samples with an IgM/IgG OD ratio of <1.2 or, negative for DENV IgM, and positive for DENV IgG [22,23]. For SARS-CoV-2 patient samples, 53 nasopharyngeal swab samples were used. All samples was confirmed as positive for SARS-CoV-2 by RT-PCR, as described previously [24] (Supplementary Information). The study protocol was approved by the Institutional Review Board at the Institute of Tropical Medicine, Nagasaki University (approval No.: 08061924–7) and the National Institute of Hygiene and Epidemiology (approval No.: IRB-VN01057–45/2016).

### 2.4. Virus Overlay Medium

Three types of overlay media to facilitate plaque formation for infectious virus detection were used in this study: (1) Avicel medium (Av), (2) Avicel and methylcellulose medium (AvMc), and (3) methylcellulose medium (Mc). To prepare the Av medium, a total of 12 g of Avicel (Asahi Kasei, Tokyo, Japan) and 9.4 g of E-MEM powder (Nissui Pharmaceutical Co., Ltd., Tokyo, Japan) were dissolved in 1 L of double distilled water (DDW) and stirred with a magnetic bar until all reagents were dissolved. To prepare the AvMc medium, a total of 5 g of Mc (Wako Pure Chemical Industries Ltd., Osaka, Japan), 6 g of Avicel, and 9.4 g of E-MEM powder were dissolved in 1 L of DDW. The Mc medium was prepared with 10 g of Mc and 9.4 g of E-MEM powder in 1 L of DDW and, the reagents were then autoclaved, cooled, and stored at 4 °C until use. For complete overlay medium, a total of 10 mL of 100 × L-glutamine (Gibco, Gaithersburg, MD, USA), 20 mL of heat-inactivated FBS (Sigma), and 31.5 mL of 7.5% sodium bicarbonate (Gibco, Gaithersburg, MD, USA) were supplemented. Each mixture was stirred for consistency prior to use.

### 2.5. Infectious Virus Particle Titration from Laboratory-Established Strains and Clinical Samples

Virus titration was performed using a conventional plaque assay [25]. BHK-21, FcγRIIA-expressing BHK-21, and Vero 9013 were used for flavivirus titration, and Vero 9013 cells were used for SARS-CoV-2 virus titration. The cells were seeded in 12-well plates (approximately 5 × 10^5^ cells per well) with 1 mL growth medium E-MEM supplemented with 10% FBS, and incubated at 37 °C in 5% CO_2_ overnight to allow the formation of a cell monolayer. Infectious cell culture fluids and patient samples were serially diluted in E-MEM from 1:10 to 1:10^6^ in 10-fold dilutions. A total of 100 µL of serially diluted sample was added to each well. At least two replicates of each sample were inoculated. The plates were then incubated at 37 °C in 5% CO_2_ for 60 min. After incubation, 2 mL of overlay medium was added to each well. For titrations of virus stock, the three types of overlay media (Av, AvMc, and Mc) were used. After 4 to 5 days of incubation, the cells were fixed with 4% paraformaldehyde for 1 h at room temperature and stained with 0.25% crystal violet (Wako Pure Chemical Industries). The number of plaques was counted with the naked eye, and plaques were captured using an Automatic Colony Counter PSF-1000 (SK-Electronics Co., Ltd., Kyoto, Japan). Virus titer was defined as the number of plaque-forming units per mL (PFU/mL). The size (mm) of 10 randomly selected plaques was measured using a Fluorescence Microscope BZ–X700 (KEYENCE, United States). Briefly, 10 plaques were randomly selected from one to three wells under the microscope with a magnifying power of 40. Each well was divided into four sections, and 2–3 plaques were randomly chosen from each section for measurement. The diameter line was placed at the major axis and measured according to manufacturer’s instructions. Flavivirus infection experiment was performed in a biosafety level 2 (BSL-2) facility, whereas SARS-CoV-2 infection experiment was performed in the biosafety level 3 (BSL-3) facility at the Institute of Tropical Medicine, Nagasaki University, according to local biosafety guidelines and regulations.

### 2.6. Data Analysis

The mean and standard deviation (SD) for virus titers and plaque size for each overlay medium in each cell line was determined by using GraphPad (GraphPad Software, La Jolla, CA, USA). Virus titer results were transformed to base-10 logarithm values for analysis. For comparison of each overlay medium, the *p*-value was calculated using two-tailed *t* tests for the viral titer and Mann–Whitney U tests for the plaque size. Statistical analysis was performed using GraphPad Prism version 6.07 (GraphPad Software, La Jolla, CA, USA).

## 3. Results

### 3.1. Infectious Virus Titration Using Laboratory-Established Flavivirus Strains and SARS-CoV-2 Strains

**Figure 1:**
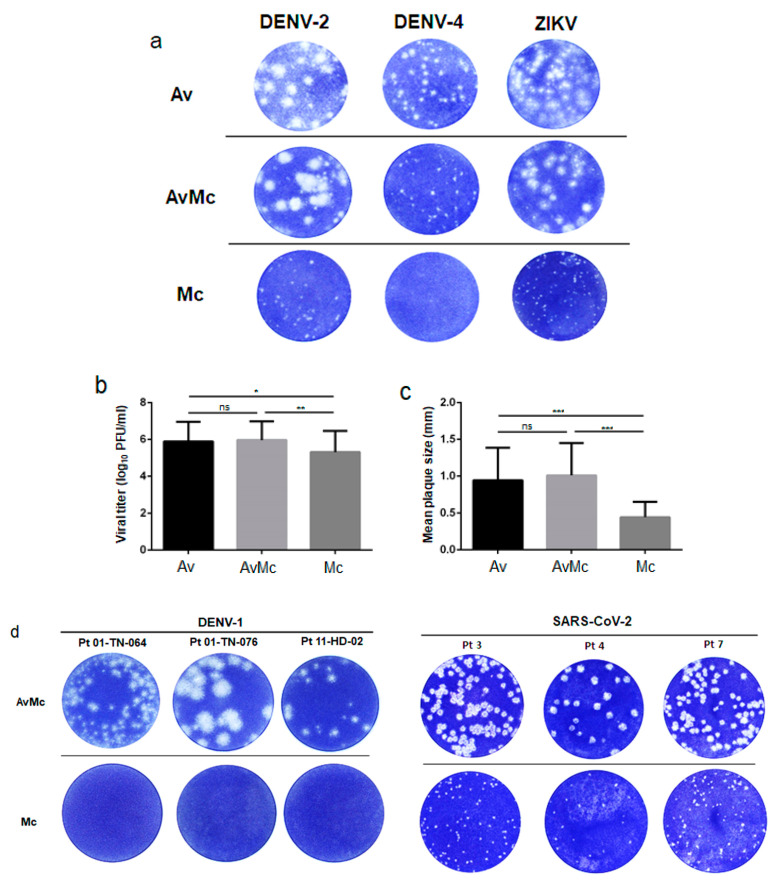
Virus titers of laboratory flavivirus strains and clinical DENV-1 and SARS-CoV-2 as determined using plaque assay. (**a**) Laboratory-established DENV-2, DENV-4, and ZIKV strains were diluted from 1:10 to 1:10^6^ and inoculated onto Vero cell monolayer in 12-well plates. Plaque formation differed significantly by number (**b**) and size (**c**) at the same dilution for each virus between Avicel medium (Av) and methylcellulose (Mc). (**d**) Infectious virus titers of six clinical samples obtained from DENV-1 and SARS-CoV-2 patients were determined using Avicel-methylcellulose medium (AvMc) and methylcellulose medium (Mc). The mean of the DENV plaque size for each overlay media (mean ± SD). Each bar shows the mean value, and the error bar represents standard deviation. Significance was determined by unpaired Student’s *t* test, ns indicates “not significant”, * *p* < 0.05, ** *p* < 0.01 and *** *p* < 0.001.

Of the 15 flavivirus strains used and among the three cell lines tested, up to one log higher virus titer was detected in 10 (66.7%), 4 (28.5%), and 14 (93.3%) flavivirus strains for BHK, Vero, and FcγR-BHK cells, respectively, when Av medium was used compared to Mc medium (Table 1, Figure 1). The log_10_ viral titer (PFU/mL) was significantly different between cultures grown in Av and Mc (*p* Av vs. Mc = 0.016, *p* AvMc vs. Mc = 0.0057, Figure 1b). Similarly, plaque size was up to five times larger when Av-containing medium was used (Table 2), and there were significant differences in plaque size when Av-containing medium was used (*p* Av vs. Mc < 0.0001, *p* AvMc vs. Mc < 0.0001, Figure 1c). Notably, while the JEV strain (OH0566) did not demonstrate higher virus titers between cell lines and overlay medium (Table 1), the plaque size was larger in Av-containing medium than in Mc medium (Table 2), demonstrating that plaque formation was larger and clearer when an Av overlay medium was used (Figure 1a). In a series of virus titrations using five types of SARS-CoV-2 strains, plaques were not detected in the two strains when Mc was used as the overlay medium (Table 3). Consistent with the results for flaviviruses, SARS-CoV-2 plaque sizes were up to two times larger when Av-containing medium was used compared to Mc medium (Table 3, (*p* AvMc vs. Mc < 0.05)).

### 3.2. Dengue Virus Titers in Serum Samples from Dengue Patients and SARS-CoV-2 Titers in Nasopharyngeal Swab Samples from COVID-19 Patients

A total of 27 samples were obtained from patients with DENV-1 infection, and virus titers in each clinical sample were determined using three different cell lines and overlay media (Table 4). In these patient samples, clear plaques were observed in 20 (74%) samples using Av or AvMc medium. In contrast, when Mc overlay medium was used, plaque formation was observed in only two samples (7%) (Table 4, Figure 1d). Using a total of 53 COVID-19 patient nasopharyngeal swab samples, clear plaques were detected in 11 (20.7%) samples using Av overlay medium. In comparison, nine (16.9%) COVID-19 samples exhibited plaques using Mc medium (Table 5, Figure 1d). The log_10_ virus titer (PFU/mL) differences between Av-containing media and Mc medium were statistically significant (*p* AvMc vs. Mc < 0.0001), the detection limit of virus titer by plaque assay was log_10_ 2.0 PFU/mL. While a total of 42 COVID-19 samples were positive for the SARS-CoV-2 genome by RT-PCR, the samples were negative for plaque formation (Supplementary Table S1). These results were consistent with the findings of other investigators that virus genome levels do not reflect infectious virus particle levels [27]. In samples obtained from DENV-1 and COVID-19 patients, plaque sizes were consistently larger and viral titer was higher when Av-containing overlay medium was used compared to Mc overlay medium (Table 4 and Table 5). In addition, the SARS-CoV-2 plaque size cultured in samples from COVID-19 patients was significantly larger when Av-containing overlay medium was used compared to Mc overlay medium (*p* < 0.05).

## 4. Discussion

Detection of infectious particles remains a challenge as live virus particles decrease concurrently with decreased virus shedding or viremia. In this study, higher virus titers and clearer plaque formation were detected in flavivirus and SARS-CoV-2 laboratory-established strains and clinical samples by a conventional plaque titration method using an improved overlay medium. As high virus titers have been associated with severe clinical outcomes [2,3,4,5], assessing infectious virus titers that better reflect the infectivity of the virus is important for determining disease pathogenesis. In addition, assessing infectivity levels that better reflect the infectiousness is important for control measures, particularly in SARS-CoV-2 infection, to accurately determine transmission capability [27].

Plaque formation using Av has also been reported for enterovirus [15], Rift Valley fever virus [16], and influenza virus [14]. However, in previous studies, focus formation was detected using the immunostaining method [28,29,30]. In the present study, a conventional plaque assay was used for all four DENV serotypes, ZIKV, JEV, and SARS-CoV-2, at various inoculation doses and in clinical samples. In addition to clearer virus plaque formation, higher virus titers were detected in both laboratory-established strains and patient samples. In the detection of plaque formation, the overlay medium prevents convectional flows in the media, allowing viruses to attach to the bottom of wells and facilitate plaque formation [14]. In this context, Avicel may be a better medium than Mc in preventing convectional flows to allow better plaque formation and, in turn, higher virus titers and larger plaque size. In some flavivirus strains, the virus titer was equal or higher under Mc overlay compared to that of Av-containing medium. This was due to the speed of virus propagation. For the strain that has high propagation speed, such as JEV and ZIKV, the plaque formation is well facilitated by an Mc overlay that is comparable to Avicel overlay. In addition, the Av medium has an advantage in the determination of plaque formation in clinical samples, as some clinical virus strains may not form clear plaques. While plaque formation may be dependent on infectivity in different cell lines, in each of the cell lines tested, larger plaques formed when Av-containing overlay medium was used compared to Mc overlay medium. In this context, clearer plaques led to the detection of higher virus titers in clinical samples, and thus, the method may better reflect virus infectivity as compared to other conventional methods. In this study, the plaque assay was used rather than immunostaining methods because viral infectivity can be detected directly with the naked eye without the staining step; hence, the plaque assay is a widely used method because of its simplicity. Some virus strains formed clearer plaques when Av-containing medium was used, and thus the medium may be suitable for the detection of a range of viral strains using plaque assays. In terms of utility, Av medium is relatively easy to use because of its low viscosity compared to Mc medium, and it can be adapted for high-throughput assays such as the 96-well and 384-well formats. In addition to clearer plaques, the incubation time can be shortened by 1 day due to the increased size and clearer plaques when Av is used. In this study, the cells were fixed after 5 days (ZIKV, JEV, YFV) or 6 days (DENV) post-infection when Mc was used as an overlay medium. In comparison, when Av was used, plaques that were large enough to be observed with the naked eye were confirmed 4 to 5 days post-infection, demonstrating that the improved method required a shorter incubation time compared to conventional medium using methylcellulose.

## 5. Conclusions

Because of clearer plaque formation, plaques were consistently detected using Avicel overlay medium even when there was no plaque formation using the conventional Mc method. One of the challenges encountered in textbook descriptions of flavivirus viremia levels and SARS-CoV-2 virus shedding is that the conventional titration method uses Mc as an overlay medium. Our results demonstrated that this classical description of virus titer may have resulted in the infectious capacity of these viruses not being robustly evaluated, because of an underestimation of the infectious particle levels. In addition, clearer plaque formation allows the use of a wider range of clinical isolates for plaque-based assays, including virological and anti-viral drug studies and neutralization antibody titration for flavivirus and SARS-CoV-2 [31,32,33,34,35], which in turn, can lead to better understanding and characterization of various clinical strains. Overall, the study results indicated that the improved virus detection method using Av as an overlay medium is rapid and practical and has the potential to serve as a useful tool for laboratory diagnosis of acute flavivirus and SARS-CoV-2 infection in virological studies.

## Figures and Tables

**Table 1:** Virus titers of 15 flavivirus strains as determined in BHK-21, Vero, and FcγR-expressing BHK cell lines.

	Log_10_ Virus Titer (PFU/mL)								
Cell Lines	BHK-21			Vero			FcγR-Expressing BHK-21		
Virus Strain	Av ^a^	AvMc ^b^	Mc ^c^	Av	AvMc	Mc	Av	AvMc	Mc
DENV-1 01-44-1 HuNIID	6.4	6.6	4.1	3.3	4.4	3.8	6.5	6.1	5.7
DENV-1 99St12A	4.4	5.5	2.5	4.5	4.7	4.8	4.9	6.1	4.6
DENV-2 DHF0663	5.4	5.1	4.4	5.4	5.6	5.0	5.9	5.8	5.2
DENV-2 TLC-30	5.0	4.8	4.5	5.0	4.7	5.0	4.5	4.8	4.0
DENV-2 08-77	4.9	4.3	5.2	5.6	5.4	5.6	6.1	5.8	5.2
DENV-2 00St22A	4.7	5.6	5.7	6.3	6.0	6.0	6.6	5.6	5.8
DENV-3 CH53469	5.8	6.7	5.4	4.6	4.3	4.1	5.9	6.9	5.8
DENV-4 SLMC318	5.7	5.6	4.9	5.4	4.1	4.4	6.1	6.0	4.3
DENV-4 TVP360	4.7	5.1	3.4	4.8	4.2	4.6	4.9	5.0	3.9
ZIKV MR766	6.2	6.8	6.9	8.2	7.6	7.1	6.9	7.4	6.1
ZIKV PRVABC59	5.5	5.9	5.0	7.1	6.8	6.7	5.1	6.1	4.4
ZIKV MRS_OPY_Martinique_PaRi_2015	5.8	6.1	5.6	7.1	6.9	6.8	5.9	6.2	4.9
ZIKV H/PF/2013	6.5	6.7	5.9	7.4	7.2	6.2	6.8	6.7	5.7
JEV OH0566	7.8	7.8	7.9	7.6	7.7	7.5	7.9	7.7	7.7
YFV 17D	6.6	6.5	5.2	5.8	6.3	6.5	6.9	6.6	4.9

^a^ Av represents Avicel medium, ^b^ AvMc represents Avicel and methylcellulose mixed medium, ^c^ represents methylcellulose medium. Plaque size was determined by random selection of 10 plaques, and values are mean +/− s.d. of each plaque diameter.

**Table 2:** Average plaque size (mm) of 15 flavivirus strains using BHK-21, Vero, and FcγR-expressing BHK cell lines.

Cell Lines	BHK-21			Vero			FcγR-Expressing BHK		
Virus Strain	Av ^a^	AvMc ^b^	Mc ^c^	Av	AvMc	Mc	Av	AvMc	Mc
DENV-1 01-44-1 HuNIID	0.8 ± 0.2	0.8 ± 0.4	0.3 ± 0.1	0.3 ± 0.2	0.3 ± 0.2	0.3 ± 0.1	1.1 ± 0.3	0.4 ± 0.1	0.4 ± 0.1
DENV-1 99St12A	1.0 ± 0.3	1.5 ± 0.3	0.4 ± 0.1	0.5 ± 0.2	0.5 ± 0.2	0.3 ± 0.1	0.9 ± 0.2	1.0 ± 0.2	0.2 ± 0.0
DENV-2 DHF0663	1.1 ± 0.3	1.1 ± 0.2	0.2 ± 0.1	1.1± 0.3	1.5 ± 0.3	0.7 ± 0.2	1.0 ± 0.4	1.1 ± 0.7	0.5 ± 0.1
DENV-2 TLC-30	1.7 ± 0.5	1.8 ± 0.6	0.5 ± 0.1	1.2 ± 0.3	1.0 ± 0.5	0.6 ± 0.1	0.8 ± 0.1	1.3 ± 0.1	0.3 ± 0.0
DENV-2 08-77	0.4 ± 0.1	0.6 ± 0.1	0.5 ± 0.1	1.0 ± 0.5	0.6 ± 0.2	0.4 ± 0.1	0.5 ± 0.1	1.4 ± 0.2	0.3 ± 0.0
DENV-2 00St22A	0.6 ± 0.1	0.8 ± 0.2	0.3 ± 0.1	1.2 ± 0.4	0.9 ± 0.4	0.6 ± 0.2	0.7 ± 0.2	1.5 ± 0.3	0.3 ± 0.1
DENV-3 CH53469	1.5 ± 0.5	1.8 ± 0.7	0.5 ± 0.1	1.3 ± 0.5	0.6 ± 0.2	0.2± 0.0	1.9 ± 0.8	1.7 ± 0.8	0.4 ± 0.1
DENV-4 SLMC318	0.9 ± 0.2	0.7 ± 0.4	0.5 ± 0.1	0.6 ± 0.1	0.7 ± 0.3	0.4 ± 0.1	0.7 ± 0.1	0.4 ± 0.3	0.4 ± 0.1
DENV-4 TVP360	0.9 ± 0.2	1.8 ± 0.6	0.4 ± 0.1	0.9 ± 0.6	0.8 ± 0.2	0.8 ± 0.3	1.0 ± 0.3	1.6 ± 0.3	0.3 ± 0.1
ZIKV MR766	0.8 ± 0.2	1.1 ± 0.6	0.4 ± 0.1	2.2 ± 0.0	1.7 ± 0.4	1.1 ± 0.2	1.2 ± 0.4	1.5 ± 0.6	0.3 ± 0.0
ZIKV PRVABC59	0.4 ± 0.1	0.6 ± 0.2	0.5 ± 0.1	1.2 ± 0.5	1.3 ± 0.3	0.5 ± 0.1	0.4 ± 0.1	0.5 ± 0.1	0.4 ± 0.2
ZIKV MRS_OPY_Martinique_PaRi_2015	0.6 ± 0.1	0.8 ± 0.2	0.4 ± 0.1	1.3 ± 0.6	1.2 ± 0.4	0.5 ± 0.2	0.5 ± 0.2	0.6 ± 0.1	0.2 ± 0.1
ZIKV H/PF/2013	0.4 ± 0.1	0.8 ± 0.3	0.4 ± 0.1	1.4± 0.9	1.4 ± 0.6	0.9 ± 0.3	0.6 ± 0.2	0.8 ± 0.3	0.2 ± 0.0
JEV OH0566	0.2 ±0.0	0.6 ± 0.1	1.0 ± 0.3	1.4 ± 0.4	1.5 ± 0.4	0.8 ± 0.1	1.3 ± 0.9	0.7 ± 0.3	0.3 ± 0.1
YFV 17D	1.2 ± 0.5	0.6 ± 0.1	0.3 ± 0.1	0.3 ± 0.1	0.7 ± 0.2	0.3 ± 0.1	1.4 ± 0.2	0.8 ± 0.3	0.4 ± 0.1

^a^ Av represents Avicel medium, ^b^ AvMc represents Avicel and methylcellulose mixed medium, ^c^ represents methylcellulose medium. Plaque size was determined by random selection of 10 plaques, and values are mean +/− s.d. of each plaque diameter.

**Table 3:** Virus titers and plaque size of four SARS-CoV-2 strains using Vero cells.

	Virus Titer (Log_10_ PFU/mL) Vero		Plaque Size	
SARS-CoV-2 Strain	AvMc ^a^	Mc ^b^	AvMc	Mc
2019-nCoV/Japan/TY/WK-521/2020	4.9	4.8	1.4 ± 0.5	0.6 ± 0.1
NGS1B	5.5	4.6	1.3 ± 0.5	0.6 ± 0.1
hCoV-19/Japan/QK002/2020 (Alpha) ^d^	3.7	ND ^c^	0.7 ± 0.2	ND
hCoV-19/Japan/TY7-501/2021(Gamma)	3.6	ND	0.8 ± 0.1	ND
hCoV-19/Japan/TY8-612/2021 (Beta)	3.5	3.2	0.7 ± 0.1	0.3 ± 0.1

^a^ AvMc indicates Avicel and methylcellulose mixed medium, ^b^ Mc indicates methylcellulose medium, ^c^ indicates that plaques were not detected, ^d^ indicates the WHO label for SARS-CoV-2 variants of concern [26]. Plaque size was determined by random selection of 10 plaques and values are mean +/− s.d. of each plaque diameter.

**Table 4:** Virus titers in primary and secondary DENV-1 infection as determined by plaque assay using Avicel and methylcellulose as the overlay medium.

Sample Code	Days ^a^	Log_10_ Genome Copies/mL	Dengue ELISA			Virus Titer (Log_10_ PFU/mL)	
			OD ^e^-IgG	OD-IgM	IgM/IgG OD Ratio	AvMc ^c^	Mc ^d^
Primary Infection							
01-TN-145	0	9.5	0.8	0.1	0.2	6.1 ± 0.03	ND
01-TN-087	1	10.2	0.5	0.1	0.2	5.4 ± 0.04	ND
29-HD-011	1	9.5	0.1	0.1	1.0	5.1 ± 0.04	ND
01-TN-067	2	7.6	0.5	0.1	0.2	6.4 ± 0.1	4.49 ± 0.03
01-TN-076	2	10.3	0.8	0.07	0.09	7.3 ± 0.1	ND
01-TN-078	2	9.2	0.9	0.06	0.06	6.2 ± 0.2	ND
01-TN-091	2	10.5	0.4	0.2	0.4	6.6 ± 0.02	ND
01-TN-040	3	8.1	0.5	0.2	0.3	6.0 ± 0.2	ND
01-TN-063	3	6.9	0.8	0.2	0.2	6.1 ± 0.07	ND
04-HD-010	7	5.2	1.8	3.5	2.0	ND ^b^	ND
08-HD-001	8	7.6	1.0	2.6	2.5	ND	ND
09-HD-014	8	5.4	1.6	3.3	2.0	ND	ND
Secondary Infection							
01-TN-088	1	10.3	1.1	0.13	8.5	6.3 ± 0.2	ND
01-TN-077	1	10.4	1.0	0.05	22.1	5.0 ± 0.1	ND
01-TN-106	3	10.0	1.5	0.16	9.9	5.3 ± 0.06	ND
11-HD-006	3	7.7	2.3	0.45	5.2	ND	ND
11-HD-012	3	8.9	1.0	0.14	7.2	5.8 ± 0.1	ND
29-HD-001	3	8.9	1.4	0.30	4.7	ND	ND
01-TN-056	3	7.6	1.5	0.13	11.5	6.1 ± 0.1	ND
01-TN-061	3	6.8	1.3	0.13	10.0	6.7 ± 0.03	ND
01-TN-064	3	7.5	1.0	0.14	6.7	6.4 ± 0.08	ND
11-HD-002	4	8.3	1.1	0.16	0.2	6.0 ± 0.05	5.5 ± 0.03
01-TN-062	5	7.2	1.6	0.13	12.8	6.1 ± 0.1	ND
01-TN-068	5	6.5	2.0	0.39	5.1	5.8 ± 0.2	ND
01-PK-119	6	5.0	1.4	0.20	7.0	ND	ND
11-HD-004	6	6.1	1.9	2.0	1.0	ND	ND
03-HD-014	8	9.5	1.9	0.79	2.4	6.5 ± 0.01	ND

^a^ Indicates days after onset of disease, ^b^ Indicates that plaques were not detected, ^c^ AvMc represents Avicel and methylcellulose mixed medium. ^d^ Mc represents methylcellulose medium. ^e^ Indicates optical density. All samples were confirmed positive for DENV by RT-PCR.

**Table 5:** Virus titers and average plaque size (mm) in COVID-19 patients as determined by using a conventional plaque assay with Avicel and methylcellulose overlay medium.

Sample No	Virus Load (log10 Genome Copies/mL)	Virus Titer (Log_10_ PFU/mL)		Plaque Size (Mean Diameters mm ± SD)	
		AvMc	Mc	AvMc	Mc
1	10.9	4.3	4.2	0.87 ± 0.34	0.29 ± 0.1
2	10.2	3.4	ND ^a^	NT	NT
3	9.9	5.1	4.9	1.0 ± 0.2	0.4 ± 0.07
4	9.7	3.8	3.6	1.3 ± 0.2	0.3 ± 0.09
5	9.7	3.2	3.1	1.4 ± 0.2	0.38 ± 0.1
6	9.5	2.6	ND	NT ^b^	NT
7	9.4	4.1	3.94	0.96 ± 0.2	0.37 ± 0.1
8	9.21	4.0	3.81	1.1 ± 0.3	0.37 ± 0.1
9	9.03	3.7	4.11	1.7 ± 0.3	0.4 ± 0.1
10	8.51	3.3	3.20	0.7 ± 0.1	0.4 ± 0.1
11	8.44	2.9	3.23	1.4 ± 0.2	0.5 ± 0.1

^a^ Indicates that plaques were not detected, ^b^ indicates “not tested”, plaque size was determined by random selection of 10 plaques and values are mean +/− s.d. of each plaque diameter.

## Data Availability

The data sets generated and/or analyzed during the current study are available from the corresponding authors on reasonable request.

## Supplementary Materials

The following are available online at https://www.mdpi.com/article/10.3390/ijerph18189845/s1, Table S1, COVID-19 clinical samples with virus titers below detection levels.
