# Supplementary material for: Discrepancies in Infectivity of Flavivirus and SARS-CoV-2 Clinical Samples: An Improved Assay for Infectious Virus Shedding and Viremia Assessment
Source: Int J Environ Res Public Health. 2021 Sep 18;18(18):9845. doi: 10.3390/ijerph18189845 (PMC8465741; doi:10.3390/ijerph18189845)
Supplement: Supplementary file 1 [file ijerph-18-09845-s001.zip › ijerph-1357255-supplementary.pdf]

Supplementary Material

Table S1. COVID-19 clinical samples with virus titers below detection levels.

Sample No	Log $_{10}$ Genome (copies/mL)	Virus titer (Log$_{10}$ PFU/mL)		Plaque size (mean diameters mm ± SD)	
		AvMc [a]	Mc [b]	AvMc	Mc
12	10.0	ND [c]	ND	NT [d]	NT
13	9.7	ND	ND	NT	NT
14	9.7	ND	ND	NT	NT
15	9.6	ND	ND	NT	NT
16	9.6	ND	ND	NT	NT
17	9.6	ND	ND	NT	NT
18	9.5	ND	ND	NT	NT
19	9.4	ND	ND	NT	NT
20	9.4	ND	ND	NT	NT
21	9.2	ND	ND	NT	NT
22	9.1	ND	ND	NT	NT
23	9.1	ND	ND	NT	NT
24	8.9	ND	ND	NT	NT
25	8.9	ND	ND	NT	NT
26	8.9	ND	ND	NT	NT
27	8.8	ND	ND	NT	NT
28	8.7	ND	ND	NT	NT
29	8.6	ND	ND	NT	NT
30	8.3	ND	ND	NT	NT
31	8.2	ND	ND	NT	NT
32	8.2	ND	ND	NT	NT
33	8.2	ND	ND	NT	NT
34	8.2	ND	ND	NT	NT
35	8.2	ND	ND	NT	NT
36	8.1	ND	ND	NT	NT
37	8.1	ND	ND	NT	NT
38	8.1	ND	ND	NT	NT
39	8.1	ND	ND	NT	NT
40	7.8	ND	ND	NT	NT
41	7.7	ND	ND	NT	NT
42	7.7	ND	ND	NT	NT
43	7.7	ND	ND	NT	NT
44	7.6	ND	ND	NT	NT
45	7.3	ND	ND	NT	NT
46	7.3	ND	ND	NT	NT
47	7.3	ND	ND	NT	NT
48	7.2	ND	ND	NT	NT
49	6.4	ND	ND	NT	NT
50	5.3	ND	ND	NT	NT
51	4.4	ND	ND	NT	NT
52	3.9	ND	ND	NT	NT
53	3.5	ND	ND	NT	NT

[a] AvMc indicates Avicel:methyl cellulose (MC) = 1:1 medium, [b] Mc indicates methyl cellulose medium, [c] Indicates that plaques were not detected. [d] Indicates “not tested”.

Supplementary Information

Methodology

(i) Real time RT-PCR for the determination of Dengue virus RNA levels

The virus genome levels for dengue virus was determined as previously reported Ito M. et al. with some modifications (J Clin Microbiol. 2004 Dec; 42(12): 5935–5937. doi: 10.1128/JCM.42.12.5935-5937.2004).

Briefly, one-step RT-PCR was performed using TaqMan Fast Virus One-Step RT-PCR Master Mix (Thermo Fisher Scientific), 0.8 μM of forward and reverse primers and 0.2 μM of probes. Next, a total of 5 μL of RNA was combined with 15 μl of master mix. The reverse transcription step was performed at 50 °C for 5 min followed by incubation at 95 °C for 20 s. Amplification included 40 cycles consisting of denaturation at 95 °C for 3 s, followed by annealing, extension and data acquisition at 60 °C for 30 s on the real time PCR machine.

(ii) Real time RT-PCR for the determination of SARS-CoV-2 RNA levels

Virus genome levels for SARS-CoV-2 was determined as previously reported (Yoshikawa R. et al. PLoS Negl Trop Dis 2020;14(11): e000855 doi: 10.1371/journal.pntd.0008855.)

Real time RT-PCR was performed by using One Step PrimeScript III RT-qPCR Mix (Takara Bio), ROX Reference Dye, 0.4 μM each of forward and reverse primers and, 0.2 μM of probes. A total of 2 μL of extracted RNA was mixed with 18 μl of the master mix. The reverse transcription step was performed at 55 °C for 10 min, followed by incubation at 95 °C for 3 min and, amplification which includes 45 cycles of denaturation at 95 °C for 15 s, followed by annealing, extension and data acquisition at 58 °C for 30 s.

The following primer and probe set was used to detect the E gene for SARS-CoV-2:

(1) E Sarbeco F: ACAGGTACGTTAATAGTTAATAGCGT

(2) E Sarbeco R: ATATTGCAGCAGTACGCACACA

(3) E Sarbeco P1: FAM-ACACTAGCCATCCTTACTGCGCTTCG-BBQ
